# No difference in the incidence or location of deep venous thrombosis according to use of pharmacological prophylaxis following total knee arthroplasty

**DOI:** 10.1186/s12891-021-04707-6

**Published:** 2021-09-23

**Authors:** Junya Itou, Umito Kuwashima, Masafumi Itoh, Ken Okazaki

**Affiliations:** Department of Orthopaedic Surgery, Tokyo Women’s Medical University, 8-1 Kawada-cho, Shinjuku-ku, Tokyo, 162-8666 Japan

**Keywords:** Deep vein thrombosis, Total knee arthroplasty, Pharmacologic prophylaxis, Ultrasonography

## Abstract

**Background:**

The incidence and characteristics of deep vein thrombosis (DVT) following total knee arthroplasty (TKA) without pharmacologic prophylaxis have not been fully investigated. This study aimed to determine whether there are any differences in the incidence, location, and characteristics of DVT following TKA with pharmacologic prophylaxis and without pharmacologic prophylaxis.

**Methods:**

A total of 156 knees were retrospectively evaluated for DVT following TKA by duplex ultrasound on postoperative day 7, after excluding 60 knees from 216 consecutive knees because of antiplatelet or anticoagulant use before surgery, history of venous thromboembolism, or bleeding risk. The 156 knees included in the analysis were divided into two groups: with pharmacologic prophylaxis (n = 79) and without pharmacologic prophylaxis (n = 77).

**Results:**

The overall incidence of DVT was 34% (54/156 knees). DVT was detected in 31.6% of knees with pharmacologic prophylaxis and in 37.6% of knees without pharmacologic prophylaxis; the difference was not statistically significant. Soleal vein thrombus was observed in 74.6% of the knees with DVT and non-floating thrombus was observed in 98.7%. There were no obvious between-group differences in thrombus characteristics such as compressibility, echogenicity, mean vein diameter, and whether the thrombus was attached to the vein wall or free-floating.

**Conclusions:**

No differences were found in the incidence, location, or characteristics of DVT following TKA with or without pharmacological prophylaxis.

## Introduction

Deep vein thrombosis (DVT) is one of the major potential complications in patients undergoing total knee arthroplasty (TKA) [1–3]. Chemoprophylaxis and/or mechanical prophylaxis (such as use of an intermittent pneumatic compression device) are recommended in the 2011 American Academy of Orthopaedic Surgeons guidelines [2, 4, 5]. Although pharmacologic intervention is known to be effective for preventing DVT [3, 5], research on pharmacologic prophylaxis has continued in efforts to reduce the incidence of DVT [1].

Risk factors for DVT following TKA have also been investigated [6]. Previous studies have suggested that ethnic differences play a role in the risk of DVT [7–9], and its incidence has been reported to be lower in Asians than in Caucasians [8–11]. Given the low incidence of DVT in Asians, routine pharmacological prophylaxis is not recommended [12–14]. There have also been several reports on the incidence of DVT following TKA without pharmacologic prophylaxis [14, 15]. Chang et al. [15] found that the overall incidence of postoperative DVT was 58.5% per knee in 253 consecutive knees that underwent TKA with mechanical prophylaxis when evaluated indirectly by computed tomography (CT) venography. In that study, symptomatic DVT occurred at a rate of 0.4% per knee. Park et al. [14] retrospectively reviewed 2891 consecutive TKAs and reported an incidence of symptomatic DVT of 0.35% per knee (n = 11) with use of mechanical prophylaxis alone. However, they did not report the overall incidence of DVT, including those that were asymptomatic. The other reports on the incidence of DVT following TKA without pharmacologic prophylaxis are relatively old and discuss postoperative management methods, such as early ambulation and exhaustive pain management, that are different from those currently used [12, 13]. Therefore, there is still limited information on the incidence of DVT without pharmacologic prophylaxis, which raises questions about whether the American Academy of Orthopaedic Surgeons guidelines should be followed [16].

Furthermore, among the studies that have investigated the difference in incidence of DVT following TKA with and without pharmacological prophylaxis, few have focused on the characteristics of thrombus. Several reports have evaluated whether the DVT was distal or proximal [15, 17], but none have assessed the distribution in the veins or the characteristics of the thrombus. Ultrasonography is a reliable method for detection of DVT [11, 18]. Compared with contrast-enhanced CT or venography, it is not only less invasive, but also provides a large amount of information on echogenicity and the compressibility of the thrombus. Also, while ultrasonography was shown to be not inferior to CT or venography in sensitivity or specificity [11], it includes information on whether a vein segment can be completely compressed under gentle probing pressure, which is the diagnostic criterion for DVT [19]. However, it remains unclear whether there are differences in the incidence and characteristics of DVT after TKA with or without pharmacological prophylaxis.

The purpose of this study was to determine whether there are differences in the incidence, location, and characteristics of DVT following TKA with pharmacologic prophylaxis versus without pharmacologic prophylaxis. We hypothesized that pharmacologic prophylaxis would have no effect on these parameters.

## Materials and Methods

### Patient eligibility and selection

**Fig. 1:**
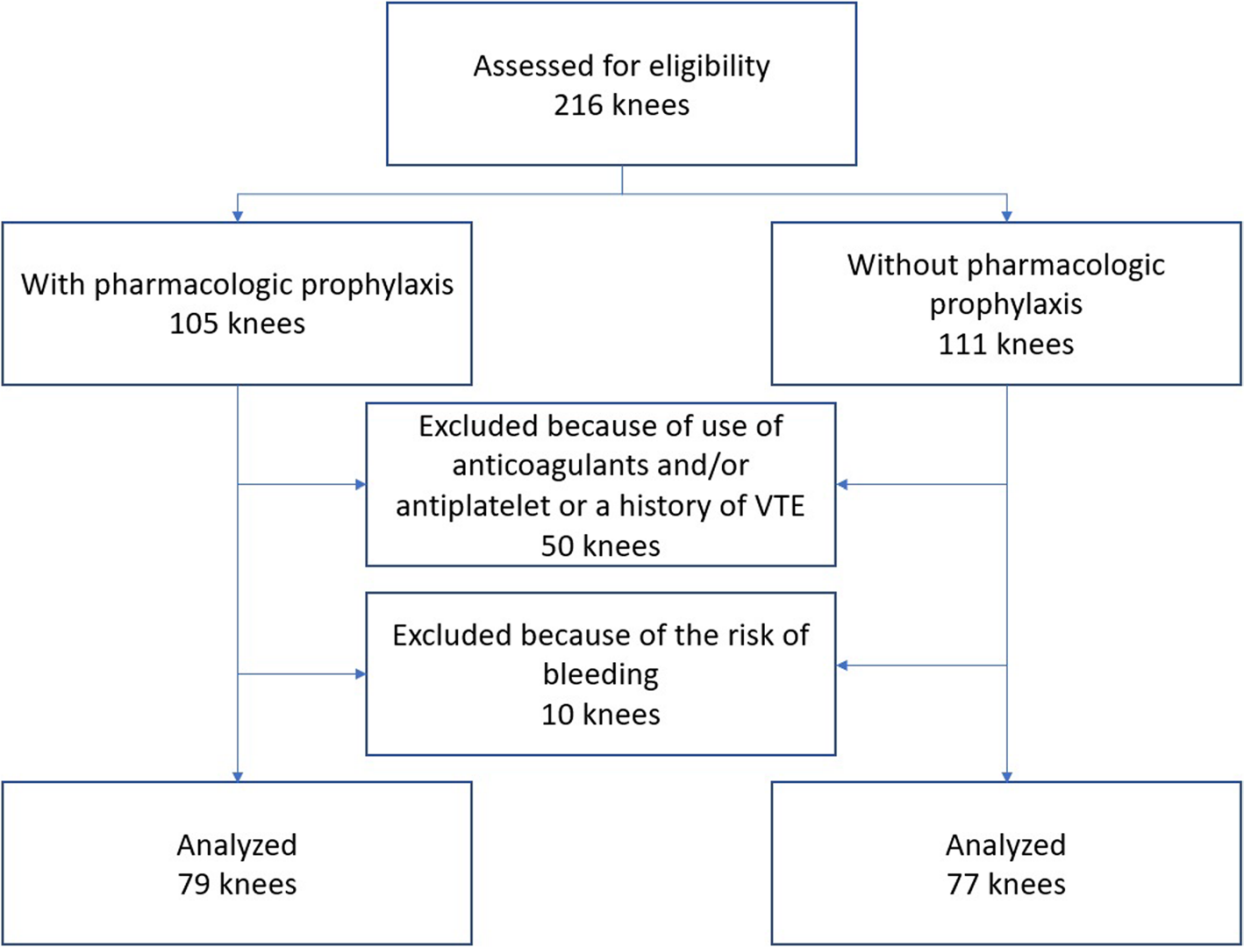
Flowchart showing the retrospectively identified cohort of patients who underwent primary TKA at our institution and the reasons for exclusion. A total of 50 knees in patients who received anticoagulants and/or antiplatelet agents or who had a history of VTE were excluded, as were 10 knees in patients with a history of VTE or bleeding risk. TKA, total knee arthroplasty; VTE, venous thromboembolism

This was a retrospective study of all 216 consecutive knees that underwent primary TKA at our institution between November 2018 and October 2020. Patients who underwent TKA in the first year of this 2-year study received a combination of physical and pharmacologic prophylaxis (November 2018–October 2019, group 1) and those who underwent TKA in the second year received physical prophylaxis only (November 2019–October 2020, group 2). Standard physical prophylaxis consisted of the use of elastic stockings and an intermittent pneumatic compression device in the early postoperative period. Pharmacologic prophylaxis consisted of administration of enoxaparin 20 mg twice daily for approximately 6 days starting 24 h postoperatively until duplex ultrasonography was performed to determine whether postoperative DVT was present. In addition, enoxaparin was administered at half dose for 14 knees because of impaired renal function or low body weight. Of the 216 consecutive knees, 60 were excluded because of 1) preoperative administration of antiplatelet agents or anticoagulants or a history of venous thromboembolism (VTE; n = 50) or 2) bleeding risk (n = 10). Bleeding risk was defined as a history of peptic ulcer or hemostatic disorder or current severe renal dysfunction. Ultimately, 155 knees with pharmacologic prophylaxis (group 1, n = 79) or without pharmacologic prophylaxis (group 2, n = 77) were included in the study (Fig. 1). There were 96 women and 34 men of mean age 70 (range, 40–86) years. There was no significant difference in preoperative height and weight, body mass index, sex ratio, or side affected between the study groups (Table 1).

**Table 1:** Patient demographics

	Group 1	Group 2	P-value
Male/female, n	21/46	13/51	0.13
Age, years	71.1 ± 7.8	69.4 ± 8.9	0.21
Height, cm	155 ± 9.1	155 ± 8.8	0.61
Weight, kg	62.7 ± 11.5	64.0 ± 13.0	0.51
Body mass index	25.7 ± 3.5	26.5 ± 4.3	0.21
Affected side, n, right/left	43/36	43/34	1.00

Data are shown as the number or the mean ± standard deviation. Group 1, patients who received pharmacologic prophylaxis. Group 2, patients who did not receive pharmacologic prophylaxis

### Surgical technique and rehabilitation interventions

The surgical procedures were performed by any of four specialist knee surgeons, with attention paid to consistency in the surgical techniques and intraoperative management across cases. The most common prostheses used were Journey II (84 knees, Smith & Nephew, Memphis, TN, USA) and Vanguard (54 knees, Zimmer, Warsaw, IN, USA); the others were Legion (2 knees, Smith & Nephew), Persona (2 knees, Zimmer), and Low Contact Stress (14 knees, DePuy, Warsaw, IN, USA). The TKA instruments were chosen according to the surgeon’s preference. All surgeries were performed via a subvastus approach. The Journey II, Legion, and Persona prostheses were cemented and the Vanguard and Low Contact Stress were cementless. A postoperative drainage tube was not always placed. A tourniquet was used in all cases, with inflation before incision and release after closing the skin. Tranexamic acid 1000 mg was administered intravenously approximately 10 min before the tourniquet was released. All surgeries were performed under general anesthesia, and additional regional anesthesia using ultrasound guidance [20] was added at the discretion of the anesthesiologist. Patients were allowed to ambulate with full weight bearing as pain permitted from the day following surgery and underwent rehabilitation without restriction of range of motion. As reported previously, allogeneic blood transfusion was performed when the hemoglobin level was below 7.0 g/dL and the patient had symptoms of anemia [21].

### Assessment for VTE

Duplex ultrasonography was performed in all patients on postoperative day 7 to determine whether postoperative DVT was present. DVT was assessed by whole leg ultrasonography [22] and the location of the thrombus was recorded. Proximal DVT was defined as DVT occurring in the popliteal vein or above. If multiple thrombi were found in the same case, each location was counted. Compressibility (firm, soft), echogenicity (hyperechoic, isoechoic, hypoechoic), vein diameter (mm), and whether the thrombus was attached to the vein wall or free-floating were also investigated. All duplex ultrasonography procedures were performed by the same team of ultrasonographers. This team consisted of non-physicians and was independent of the surgeon who performed the surgery. If pulmonary thromboembolism was suspected based on clinical findings, contrast-enhanced CT was added. Patients who were confirmed to have DVT on duplex ultrasonography were treated with apixaban. If DVT was not present on duplex ultrasonography, pharmacologic prophylaxis was discontinued.

### Assessment for complications

Complications occurring up to 6 months postoperatively were retrospectively assessed by review of the patients’ medical records. Complications related to pharmacological prophylaxis were defined as major bleeding (e.g., death or a life-threatening clinical event), minor bleeding (an overt bleeding episode that did not meet the criteria for major bleeding) [23], and additional wound procedures. Changes in hemoglobin levels recorded during the perioperative period were evaluated to assess the degree of anemia. In addition, postoperative complications of interest were assessed, such as superficial and deep wound infections, fractures, and revision.

### Ethical approval

This study was approved by the institutional ethics committee of Tokyo Women’s Medical University (Approval No. 4952). Informed consent was obtained via the opt-out method. All procedures involving human participants were in accordance with the ethical standards of the 1964 Helsinki Declaration and its later amendments.

### Statistical analysis

Categorical variables were examined using the chi-squared and Cochran-Armitage tests. Continuous variables were assessed using the two-tailed t-test or Mann–Whitney U test as appropriate. All statistical analyses were performed using JMP software version 15 (SAS Institute Inc., Cary, NC, USA). A p-value < 0.05 was considered to indicate statistical significance.

## Results

### Incidence of DVT

The overall incidence of DVT was 34.6% (54/156 knees; Table 2). DVT was detected in 31.6% of knees in group 1 and in 37.6% of those in group 2, with no statistically significant difference between the groups (p = 0.50).

**Table 2:** Incidence of deep vein thrombosis in the two study groups

	Group 1	Group 2	P-value
DVT (knees), n (%)	24 (31.6)	29 (37.6)	0.50

Data are shown as the median (percentage). Group 1, patients who received pharmacologic prophylaxis. Group 2, patients who did not receive pharmacologic prophylaxis

### Distribution and characteristics of DVT

**Fig. 2:**
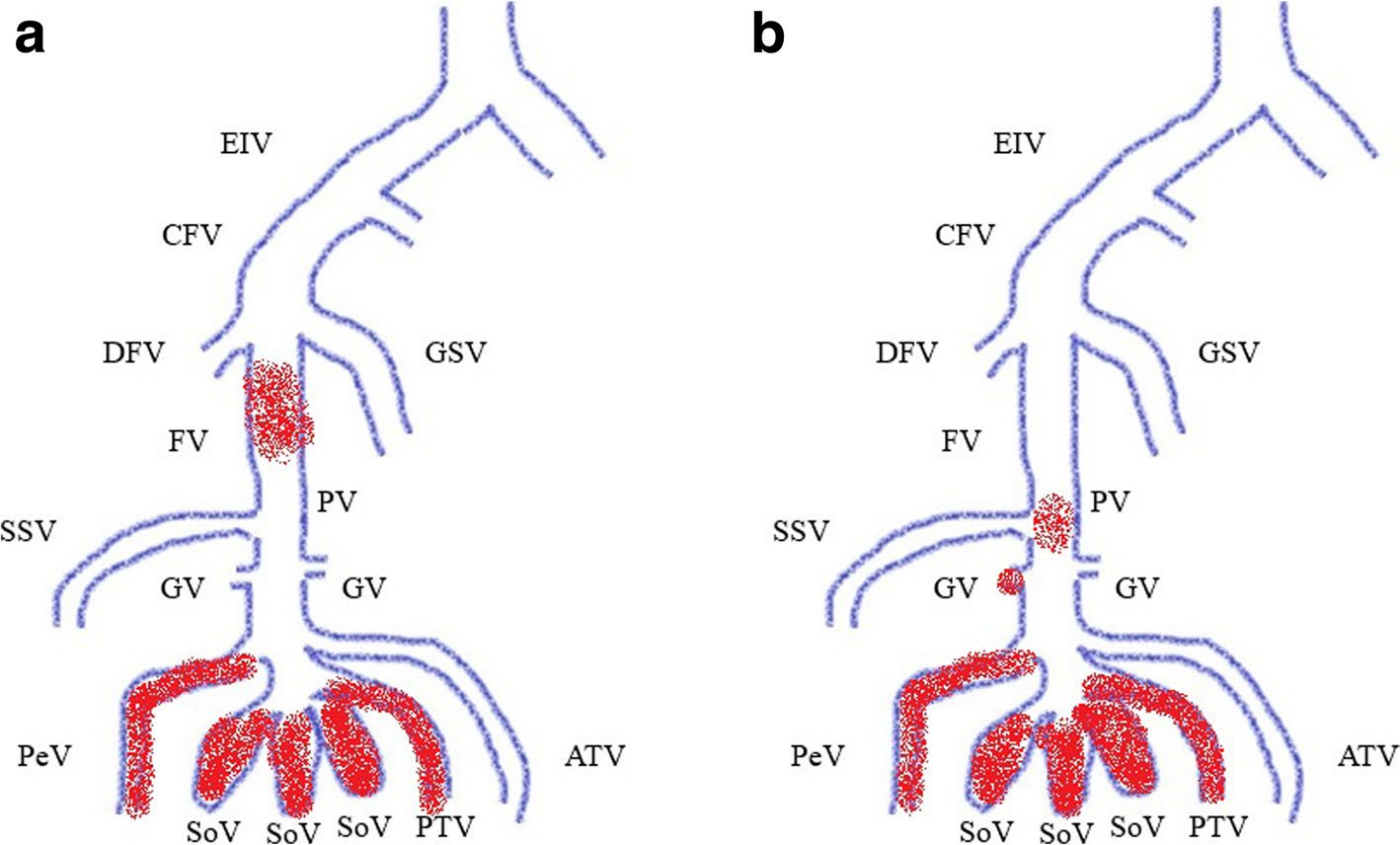
Distribution of DVT in leg veins. a Patients who received pharmacologic prophylaxis (group 1). b Patients who did not receive pharmacologic prophylaxis (group 2). In group 1, in addition to distal DVT, there were 2 knees with non-floating proximal thrombi in the femoral vein. In group 2, only distal thrombi were observed. ATV, anterior tibial vein; CFV, common femoral vein; DFV, deep femoral vein; DVT, deep vein thrombosis; FV, femoral vein; GSV, great saphenous vein; GV, gastrocnemius vein; PeV, peroneal vein; PTV, posterior tibial vein; PV, popliteal vein; SoV, soleal vein; SSV, small saphenous vein.

DVT was soleal vein thrombus in 74.6% of cases and non-floating thrombus in 98.7% (Fig. 2a, b). In group 2, there was one case (1.3%) of floating thrombus located in the popliteal vein that disappeared after 2 weeks with postoperative anticoagulation. Details of the postoperative DVTs are shown in Table 3. All cases of thrombus in the contralateral unaffected leg were found in the soleal vein, with no significant between-group difference in distribution (Table 3). In addition, multiple thrombi were identified in 18 patients, 7 in group 1 and 11 in group 2.

**Table 3:** Comparison of the distribution of DVT in the veins of the lower extremities in the two study groups

	Location	Group 1, n	Group 2, n
Proximal	CFV	0	0
	FV	2	0
	DFV	0	0
	PV	0	1
Distal	PTV	2	4
	PeV	3	5 (2)
	ATV	0	0
	SoV	22 (5)	26 (6)
	GV	0	1

Group 1, patients who received pharmacologic prophylaxis. Group 2, patients who did not receive pharmacologic prophylaxis. ATV, anterior tibial vein; CFV, common femoral vein; DFV, deep femoral vein; DVT, deep vein thrombosis; FV, femoral vein; GV, gastrocnemius vein; PeV, peroneal vein; PV, popliteal vein; PTV, posterior tibial vein; SoV, soleal vein ( ), thrombus in the contralateral knee

There were no obvious between-group differences in thrombus characteristics when evaluated by compressibility (firm or soft), echogenicity (hyperechoic, isoechoic, or hypoechoic), mean vein diameter, and whether the thrombus was attached to the vein wall or free-floating (Table 4).

**Table 4:** Characteristics of the two groups on duplex ultrasonography

Variable	Group 1	Group 2	*P* value
Compressibility (firm/soft)	21/13	36/9	0.07^a^
Echogenicity (hyperechoic/isoechoic/hypoechoic)	1/14/19	2/17/26	0.91^b^
Mean vein diameter (mm)	5.6 ± 1.7	5.4 ± 1.5	0.49^c^
Attachment of thrombus to vein wall	Attached, 34; free-floating, 0	Attached, 44; free-floating, 1	1.00^a^

Data are shown as the number or as the mean ± standard deviation. Group 1, patients who received pharmacologic prophylaxis. Group 2, patients who did not receive pharmacologic prophylaxis. ^a^Chi-squared test; ^b^Cochran-Armitage test; ^c^Mann–Whitney U test

### Complications

Pharmacologic prophylaxis with enoxaparin was discontinued in 5 knees (6.4%) in group 1 because of worsening swelling and prolonged bleeding. One further knee (1.2%) in group 1 required additional suturing using skin staplers. During the perioperative period, the hemoglobin level decreased by 3.1 ± 1.1 g/dL in group 1 and by 3.0. ± 1.0 g/dL in group 2 (p = 0.80; Table 5). In group 1, there was one case of pulmonary thromboembolism in a patient who also had a distal DVT. Intervention by cardiologists avoided a fatal outcome and the patient was able to be discharged home. Furthermore, no other complications, such as infection and revision, were seen in either group.

**Table 5:** Decrease in hemoglobin level during the perioperative period

	Group 1	Group 2	P-value
Decrease in hemoglobin level, g/dL,	3.1 ± 1.1	3.0 ± 1.0	0.80

Group 1, patients who received pharmacologic prophylaxis. Group 2, patients who did not receive pharmacologic prophylaxis

### Results of statistical effects

A post hoc analysis of the correlation between the two groups was performed using G*Power (Universität Kiel, Kiel, Germany). The statistical power was 0.87 with an effect size of 0.5, an alpha value of 0.05, and a sample size of 79 (group 1) and 77 (group 2).

## Discussion

At our institution, enoxaparin was routinely used as pharmacologic prophylaxis for DVT following TKA until October 2019 when a preliminary survey unexpectedly showed the incidence of DVT to be approximately 30% in these patients. After pharmacologic prophylaxis was stopped, we conducted the present retrospective observational study to obtain information on the incidence and characteristics of DVT in patients who undergo TKA without pharmacologic prophylaxis. Our hypothesis was that there would be no difference in the incidence, location, or characteristics of DVT following TKA between those with and without pharmacologic prophylaxis. Our findings were as follows: (1) the incidence of DVT in patients who underwent primary TKA without pharmacologic prophylaxis was 37.6%, which was not significantly different from the 31.6% incidence of DVT in patients who received concomitant pharmacologic prophylaxis; (2) most of the DVTs occurred in the soleal vein, with no significant in distribution between the two groups; and (3) a total of 6 knees (7.6%) had complications, most of which involved interruption of pharmacologic prophylaxis due to swelling and prolonged bleeding.

In 2008, a randomized double-blind study with 396 Japanese patients undergoing primary TKA compared the incidence of VTE in those administered enoxaparin 40 mg/day with that in those administered a placebo [17]. It found that the incidence of VTE was 29.8% in the enoxaparin group and 60.8% in the placebo group. Even taking into account the possibility of differences in patient background factors, various additional factors may have contributed to the decline in the incidence of VTE. These include the development of surgical techniques for TKA, changes in anesthesia methods such as ultrasound guidance in regional anesthesia [20], increased use of a multimodal analgesic pathway for TKA [24], and changes in rehabilitation programs such as early ambulation and more widespread education of medical staff and patients about VTE [1]. In our study, which was performed in an era of modern surgical and anesthesia techniques and postoperative management, the incidence of DVT without pharmacologic prophylaxis was 37.6%. This figure is comparable with that in a report on patients receiving TKA without pharmacologic prophylaxis in 2016 [15].

To the best of our knowledge, there have been no previously published comparisons of the location or quality of thrombus in patients undergoing current TKA with or without pharmacologic prophylaxis. It was widely known that DVT develops in the soleal vein because of stasis of blood flow [1, 25]. Abe et al. investigated DVT after TKA using ultrasonography and found that all cases of DVT occurred in the soleal vein [1]. However, the results of the present study indicate that postoperative DVT is prone to occurring in the posterior tibial vein and peroneal vein as well as in the soleal vein. Therefore, DVT investigations should include not only the soleal vein but also other distal veins.

The ultrasound characteristics of DVT have been reported in the past [19]. A study described acute clots as firm or slightly deformed by compression, hypoechoic or isoechoic in echogenicity, present in a vein with an enlarged diameter, and attached to the vein wall [26]. However, in many cases, the findings overlap, making assessment of fresh thrombus difficult. Although there was no obvious difference in echogenic findings between the two groups in this study, further studies on the impact of pharmacologic prophylaxis on thrombus quality are warranted.

This study had several limitations. First, it had a retrospective design, which means that there was a possibility of patient selection bias. Although there was no significant difference in background factors between the two study groups, we cannot rule out the possibility that the incidence of VTE may have been affected by underlying diseases, including cancer [27]. Second, the sample size small. Nevertheless, the statistical power was relatively high. Third, only enoxaparin was used for pharmacologic prophylaxis. In recent years, the trend for prophylactic anticoagulation has been toward oral anticoagulants such as apixaban, rivaroxaban, dabigatran, and edoxaban [5]. However, enoxaparin is still widely used as a prophylactic anticoagulant, and its non-inferiority when used as a control has been confirmed in many randomized controlled trials [23]. Fourth, DVTs that occurred after postoperative day 7 might have been missed. In the multinational Global Orthopaedic Registry study, which included 8326 patients, the mean time to VTE after TKA was 9.7 ± 14.1 days [28]. Meanwhile, Yamaguchi et al. [29] found that symptomatic DVT peaked 4 days after TKA surgery. In another study by Song et al. [30], assessments for VTE after TKA were performed on postoperative days 3–7. Therefore, examination on postoperative day 7 can be considered appropriate. Fifth, DVT was detected by duplex ultrasonography. Unlike venography, duplex ultrasonography is minimally invasive and is considered the gold standard to date [25, 27]. Contrast-enhanced CT has been reported [15], but it is difficult to use when patients have impaired renal function or when evaluating the area around the knee due to halation from the artificial joint. Sixth, the incidence of symptomatic DVT was not assessed in this study. Assessing the symptoms of DVT from the clinical findings after TKA surgery is reported to be challenging [11], however, and the most important reason for not assessing incidence in the present study was that the study purpose was to determine whether there are differences of DVT following TKA with pharmacologic prophylaxis versus without pharmacologic prophylaxis. Finally, ethnic and cultural differences may have affected the results. All patients in this study were Asian, so care should be taken when extrapolating the results to other populations and institutions.

## Conclusions

This study retrospectively investigated the incidence of perioperative VTE, the distribution and characteristics of DVT, and complications of pharmacological prophylaxis in patients undergoing current TKA. The overall incidence of VTE was 34.6%. DVT was detected in 31.6% of knees in patients who received pharmacological prophylaxis and in 37.6% of knees in patients who did not, and the difference was not statistically significant. Most of the DVTs occurred in the soleal vein regardless of whether or not pharmacological prophylaxis was provided. There were no significant differences in properties such as thrombus compressibility and echogenicity between the study groups, but further studies are warranted. Complications of pharmacological prophylaxis were comparable with those reported previously, suggesting that it is important to consider the risk in each individual case.

## Data Availability

The datasets used and/or analyzed during the present study are available from the corresponding author on reasonable request.

## Abbreviations

DVT: Deep vein thrombosis
TKA: Total knee arthroplasty
CT: Computed tomography
VTE: Venous thromboembolism

## References

[1] Abe K, Yuda S, Yasui K, Okubo A, Kobayashi C, Muranaka A (2017). Soleal vein dilatation assessed by ultrasonography is an independent predictor for deep vein thrombosis after major orthopedic surgery. J Cardiol.

[2] Lieberman JR (2018). Deep vein thrombosis prophylaxis: State of the art. J Arthroplasty.

[3] Santana DC, Emara AK, Orr MN, Klika AK, Higuera CA, Krebs VE (2020). An update on venous thromboembolism rates and prophylaxis in hip and knee arthroplasty in 2020. Medicina (Kaunas).

[4] Mont MA, Jacobs JJ, Boggio LN, Bozic KJ, Della Valle CJ, Goodman SB (2011). Preventing venous thromboembolic disease in patients undergoing elective hip and knee arthroplasty. J Am Acad Orthop Surg.

[5] Trivedi NN, Fitzgerald SJ, Schmaier AH, Wera GD (2019). Venous thromboembolism chemoprophylaxis in total hip and knee arthroplasty: a critical analysis review. JBJS Rev.

[6] Wakabayashi H, Hasegawa M, Niimi R, Yamaguchi T, Naito Y, Sudo A (2017). The risk factor of preoperative deep vein thrombosis in patients undergoing total knee arthroplasty. J Orthop Sci.

[7] Lee WS, Kim KI, Lee HJ, Kyung HS, Seo SS (2013). The incidence of pulmonary embolism and deep vein thrombosis after knee arthroplasty in Asians remains low: a meta-analysis. Clin Orthop Relat Res.

[8] Owens JM, Bedard NS, Dowdle SB, Gao Y, Callaghan JJ (2018). Venous thromboembolism following total knee arthroplasty: Does race matter?. J Arthroplasty.

[9] Zeng Y, Si H, Wu Y, Yang J, Zhou Z, Kang P (2018). The incidence of symptomatic in-hospital VTEs in Asian patients undergoing joint arthroplasty was low: a prospective, multicenter, 17,660-patient-enolled cohort study. Knee Surg Sports Traumatol Arthrosc.

[10] Kanchanabat B, Stapanavatr W, Meknavin S, Soorapanth C, Sumanasrethakul C, Kanchanasuttirak P (2011). Systematic review and meta-analysis on the rate of postoperative venous thromboembolism in orthopaedic surgery in Asian patients without thromboprophylaxis. Br J Surg.

[11] Ngarmukos S, Kim KI, Wongsak S, Chotanaphuti T, Inaba Y, Chen CF, et al. Asia-Pacific venous thromboembolism consensus in knee and hip arthroplasty and hip fracture surgery: Part 1. Diagnosis and risk factors. Knee Surg Relat Res. 2021;19, 33(1):18. 10.1186/s43019-021-00099-y PMC8214263 34147134

[12] Bin Abd Razak HR, Soon AT, Dhanaraj ID, Tan AH (2012). Incidence of clinically significant venous thromboembolic events in Asian patients undergoing total knee arthroplasty without anticoagulation. J Arthroplasty.

[13] Kim YH, Kulkarni SS, Park JW, Kim JS (2015). Prevalence of deep vein thrombosis and pulmonary embolism treated with mechanical compression device after Total knee arthroplasty in Asian patients. J Arthroplasty.

[14] Park YG, Ha CW, Lee SS, Shaikh AA, Park YB (2016). Incidence and fate of “symptomatic” venous thromboembolism after knee arthroplasty without pharmacologic prophylaxis in an Asian population. J Arthroplasty.

[15] Chang MJ, Song MK, Kyung MG, Shin JH, Chang CB, Kang SB (2018). Incidence of deep vein thrombosis before and after total knee arthroplasty without pharmacologic prophylaxis: a 128-row multidetector CT indirect venography study. BMC Musculoskelet Disord.

[16] Farfan M, Bautista M, Bonilla G, Rojas J, Llinas A, Navas J (2016). Worldwide adherence to ACCP guidelines for thromboprophylaxis after major orthopedic surgery: A systematic review of the literature and meta-analysis. Thromb Res.

[17] Fuji T, Ochi T, Niwa S, Fujita S (2008). Prevention of postoperative venous thromboembolism in Japanese patients undergoing total hip or knee arthroplasty: two randomized, double-blind, placebo-controlled studies with three dosage regimens of enoxaparin. J Orthop Sci.

[18] Zhang Y, Xia H, Wang Y, Chen L, Li S, Hussein IA (2019). The rate of missed diagnosis of lower-limb DVT by ultrasound amounts to 50% or so in patients without symptoms of DVT: A meta-analysis. Medicine (Baltimore).

[19] Maufus M, Elias A, Barrellier MT, Pernod G (2018). Diagnosis of deep vein thrombosis recurrence: Ultrasound criteria. Thromb Res.

[20] Kampitak W, Tanavalee A, Ngarmukos S, Tantavisut S (2020). Motor-sparing effect of iPACK (interspace between the popliteal artery and capsule of the posterior knee) block versus tibial nerve block after total knee arthroplasty: a randomized controlled trial. Reg Anesth Pain Med.

[21] Shin JM, Hong SJ, Choi KH, Shin SI, Lee DK, Lee SS (2019). Low relative muscle volume: Correlation with prevalence of venous thromboembolism following total knee arthroplasty. PLoS One.

[22] Guyatt GH, Sharma AM (2012). Methodology for the development of antithrombotic therapy and prevention of thrombosis guidelines: Antithrombotic Therapy and Prevention of Thrombosis, 9th ed: American College of Chest Physicians Evidence-Based Clinical Practice Guidelines. Chest.

[23] Colwell CW, Collis DK, Paulson R, McCutchen JW, Bigler GT, Lutz (1998). Comparison of enoxaparin and warfarin for the prevention of venous thromboembolic disease after total hip arthroplasty. Evaluation during hospitalization and three months after discharge. J Bone Joint Surg Am.

[24] Elmallah RK, Chughtai M, Khlopas A, Newman JM, Stearns KL, Roche M (2018). Pain control in total knee arthroplasty. J Knee Surg.

[25] Ohgi S, Tachibana M, Ikebuchi M, Kanaoka Y, Maeda T, Mori T (1998). Pulmonary embolism in patients with isolated soleal vein thrombosis. Angiology.

[26] Gornik HL, Sharma AM (2014). Duplex ultrasound in the diagnosis of lower-extremity deep venous thrombosis. Circulation.

[27] Di Nisio M, van Es N, Büller HR (2016). Deep vein thrombosis and pulmonary embolism. Lancet.

[28] Warwick D, Friedman RJ, Agnelli G, Gil-Garay E, Johnson K, FitzGerald G (2007). Insufficient duration of venous thromboembolism prophylaxis after total hip or knee replacement when compared with the time course of thromboembolic events: findings from the Global Orthopaedic Registry. J Bone Joint Surg Br.

[29] Yamaguchi T, Hasegawa M, Niimi R, Sudo A (2010). Incidence and time course of asymptomatic deep vein thrombosis with fondaparinux in patients undergoing total joint arthroplasty. Thromb Res.

[30] Song K, Xu Z, Rong Z, Yang X, Yao Y, Shen Y (2016). The incidence of venous thromboembolism following total knee arthroplasty: a prospective study by using computed tomographic pulmonary angiography in combination with bilateral lower limb venography. Blood Coagul Fibrinolysis.

